# Economic Burden of Inappropriate Antibiotic Use for Prophylactic Purpose in Shiraz, Iran

**Published:** 2011-04-01

**Authors:** N Hatam, M Askarian, A R Moravveji, O Assadian

**Affiliations:** 1 Department of Health Management, Shiraz University of Medical Sciences, Shiraz, Iran; 2 Department of Community Medicine, Shiraz University of Medical Sciences, Shiraz, Iran; 3 Department of Hygiene and Medical Microbiology, Medical University of Vienna, General Hospital Vienna, Vienna, Austria

**Keywords:** Antibiotic, Prophylaxis, Economic Burden, Iran

## Abstract

**Background:**

Because economic data on the prophylactic usage of antibiotic in Iran are scant, we have conducted a cross-sectional study with provider perspective to measure costs and appropriate use of antibiotics in surgical wards of 6 training hospitals affiliated to Shiraz University of Medical Sciences (SUMS), Iran.

**Methods:**

Over a six-month period 1,000 consecutive patients undergoing surgical operation were enrolled and information on prophylactic antibiotic administration was collected. The information included basic patient's demographic data, types of surgery, category of antibiotic, dosage, dosage intervals, route of administration, number of doses, initiation times and duration of administration. In order to determine the agreement between prescribed antibiotics and medical indication, the American Society of Health-System Pharmacists (ASHP) guidelines were applied.

**Results:**

Nine hundred and ninety three out of 1,000 patients (99.3%) had received at least one antibiotic and 908 patients (91.4 %) received antibiotics because of a medical indication. Five out of 913 patients who had indications for antibiotic prophylaxis did not receive any antibiotic. Antibiotics were prescribed for 85 out of 87 (98%) procedures in which an antibiotic was not indicated. The average cost of antibiotic prescription per surgical procedure was 786,936 Iranian Rials (corresponding to 99.60 USD or €82.90). The most frequent prescribed antibiotic was cefazoline adding 53.3% of the total cost of antibiotics. In total, 36,516,190 Iranian Rials (corresponding to 4,622.95 USD or €3,845.20) were spent for cefazoline alone.

**Conclusion:**

The results of this study showed that all surgical patients received at least one antibiotic as prophylaxis for any infection in the surgical site. Our results indicate over- and misuse of antibiotics in Iran leading to a great amount of economic burden, since in 98% of all procedures, antibiotics were used inappropriately.

## Introduction

Without any doubt in course of infectious diseases, antibiotics can have an important role in global reduction of morbidity and mortality. However, if mis- or overused, they would result into an emergence for resistance to bacteria and lead to unnecessary costs.[1] Bacterial resistance is associated with increased morbidity and prolonged hospital stay, hence, contributes to a greater indirect costs.

In several hospital settings in the US and Europe, antibiotics were shown as the second prescribed therapeutic drugs. 60-90% of patients receive antibiotics, and in 40% of the cases, prescriptions are often without any laboratory confirmation or have any indication for antibiotic use. It was shown that the rate of antibiotic misuse are higher in surgical departments than in internal medicine ones, and were mostly for prophylaxis than treatment.[2]

In developing countries, antibiotics are the largest single group of drugs purchased by patients. At the same time, in many developing countries, the availability and use of antibiotics are poorly controlled and of course, it has been demonstrated that a restriction policy can be effective in decreasing the antibiotic use and increasing the rational prescription.[3] Misuse of an antibiotic is a multifactorial problem such as economic incentives, social norms that govern interactions between patients and physicians and between physicians too.[4] In recent years, the lack of governmental support to control the pharmaceutical industries has resulted in an increase in medication prices per capita in Iran. On the other hand, the average of therapeutics’ usage in Iran is 2 to 4 times higher than in Europe or the US. The most frequently prescribed drugs are acetaminophen and antibiotics for treatment of upper respiratory infections.[5]

There may be many explanations for this trend, including the patients’ wrong attitude in using excessive drugs and doctors’ tendency towards satisfaction of their patients though prescribing the requested medications.[5] On the other hand, some studies revealed that the fear of increased costs should not limit the attempts to improve the appropriateness of an antibiotic administration.[6] These factors may also lead to an improvement in antibiotics prescription, especially in hospital settings.

Because of mis- and overuse of antibiotics to prevent any infection in the surgical site in Shiraz, southern Iran[7][8][9] and also because of shortage of economical data on expenses of the prophylactic use of antibiotics in Iran, this cross-sectional study was performed to measure the costs and appropriate use of antibiotics in surgical wards of 6 teaching hospitals affiliated to Shiraz University of Medical Sciences (SUMS), Iran.

## Materials and Methods

From February to July 2004 in surgical wards of 6 teaching hospitals affiliated to Shiraz University of Medical Sciences, Iran, similar to previous studies,[7][8][9] 1,000 consecutive patients undergoing surgical operation were enrolled. Information on prophylactic antibiotic administration by referring to medical records and filling out data forms was collected. The collected information included hospital’s specifications, basic patient’s demographic data, type of surgery, and detailed antibiotic prescription (category of antibiotic, dosage, dosage intervals, route of administration, and number of doses, initiation times and duration of administration).

In order to determine the agreement between prescribed antibiotics and surgical treatment, the American Society of Health-System Pharmacists (ASHP) guidelines were applied.[10] The collected data was double-checked by a medical expert in this field (infection control and pharmacology). By calculating direct costs of all antibiotics used and comparing the direct cost of ASHP guideline recommendation for each patient, the difference between real cost and recommended cost was defined as extra cost. On July 31st 2004, 1 Iranian Rial corresponded to 0.0001266 USD or €0.0001053, respectively.

## Results

Among 1,000 consecutive surgical patients, 120 (12.0%) were in departments of Cardiosurgery, 112 (11.2%) in General/Gastrointestinal, 110 (11.0%) in Neurosurgery, 111 (11.1%) in Gynecology, 110 (11.0%) in Ophthalmology, 115 (11.5%) in Orthopedy, 111 (11.1%) in ENT (Ear, Nose, Throat), and 110 (11.0%) in Urology Department. One-hundred and one patients (10.1%) were transplanted patients, among them 23 (2.3% of all patients) were kidney, and 78 (7.8% of all patients) liver transplanted ones.

**Fig. 1:**
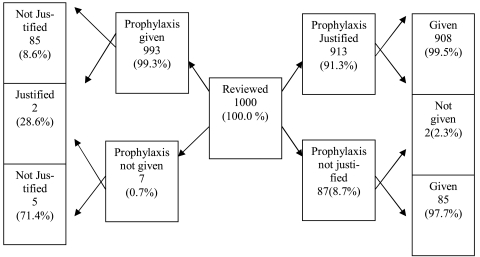
Breakdown of procedures, according to justification and actual provision of prophylaxis

The patient’s age range was 86 years (1 to 87) (mean: 40±22). More than half of the patients were male (56%), and most of the procedures (91.4%) were elective. Almost all patients (993/1000; 99.3%) had at least one prescribed antibiotic, and 908 patients (91.4%) received antibiotics because of a medical indication. Five of 913 patients who had indications for antibiotic prophylaxis did not receive any antibiotic. However, an antibiotic was prescribed for 85 out of 87 (98%) procedures in which an antibiotic was not indicated (Figure 1).

The costs of antibiotics used for various types of surgeries are shown in Diagram 1. The average cost of antibiotic prescription per surgical procedure was 786,936 Iranian Rials (corresponding to 99.60 USD or €82.90). The most frequent prescribed antibiotic was cefazoline (Table 1) which was used in 67.4% of procedures and added 53.3% of the total cost of antibiotics. In total, 36,516,190 Iranian Rials (corresponding to 4,622.95 USD or €3,845.20) were spent for cefazoline. In Table 1: Bring all cost as USD, B) Information of "the most and least usage by ward", does not give any valuable information. Generally, the highest usage of antibiotics was found in Cardio-thoracic Surgery Ward accounting for 27% of the total cost of antibiotics during the observation period.

**Table 1:** Antibiotics used and their costs in various types of surgeries

**Rank**	**Antibiotic**	**Most of use**		**Least of use**		**Total price^a^**	**Percent of total cost (%)**
		**cost/ USD**	**Ward^b^**	**cost/ USD**	**Ward^b^**		
1	Cefazolin	1550	Cardiothoracic	29	Ophthalmology	4622	53.3
2	Amikacin	635	Cardiothoracic	1	General & Gastrointestinal	700	8
3	Metronidazole	426.60	General & Gastrointestinal	.70	Neurosurgery	533	6.1
4	Ceftriaxone	303.79	Transplantation	3.89	Orthopedics	762.9	8.8
5	Clindamycin	297	Ceftriaxone	30.07	ENT	384.7	4.4
6	Cefalexin	184.25	Ceftriaxone	24	General & Gastrointestinal	611.4	7.05
7	Vancomycin	142	Neurosurgery	(only use in this ward)		142	1.6
8	Gentamycin	132.9	Orthopedics	0.68	Transplantation	480	5.5
9	Ciprofloxacin	71	Urology	0.50	Cardiothoracic	104	1.2
10	Ampicillin	69.26	General & Gastrointestinal	0.96	Neurosurgery	103.5	1.1
11	Cefixime	64	Urology	1.55	Cardiothoracic	88	1
12	Amoxicillin	44.2	ENT	10.36	General & Gastrointestinal	54.5	0.6
13	Chloramphenicol	36.66	cardiology	22.13	Ophthalmology	88	6.7
14	Penicillin	11.34	Orthopedics	2.48	General & Gastrointestinal	13.8	0.1
15	Cloxacillin	8.08	Neurosurgery	0.66	General & Gastrointestinal	8.75	0.1
16	erythromycin	1.15	General & Gastrointestinal	0.27	Ophthalmology	2.47	0.0
	Total cost			8666.25			

^a^ MAX: Cefazolin 4622 MIN: Erythromycin 2.47 Ceftriaxone 764.23 Cloxacillin 8.75 Amikacin 700 Amoxicillin 54.56

^b^ Most of antibiotic cost: Cardiothoracic Ward 2356.13, Least of antibiotic cost : Ophthalmology Ward 159.85

The quantity/ (DDD/strength)/ 1000 patients= number of DDDs per user per year which was as follows: Cefazolin=7.8175 g, Gentamicin=0.11 g, Amikacin=0.3477 g, Cephalexin=1.181 g, Ampicillin=0.06 g, Ceftriaxone=0.378 g, Cefixime=0.031 g, Chloramphenicol=0.15 g, Ciprofloxacin=0.311 g, Metronidazole=0.23 g, Amoxicillin=0.06 g, Cloxacillin=0.01 g, Clindamycin=0.02 g, Erythromycin=0.004 g, Vancomycin=0.017 g and Penicillin G=97500 units.

## Discussion

The results of this study showed that almost all surgical patients received at least one antibiotic as prophylaxis for any infection in the surgical sites. Although this reflects the surgeons’ awareness of the value of antibiotics in prevention of surgical site infections, our results also showed the overuse of antibiotics in Iran, since in 98% of all procedures antibiotics were used inappropriately. This finding is consistent with other studies conducted in average income countries.[11][12]

Bugnon-Reber and colleagues pointed out that the lack of indication is a popular reason for inappropriate use of antibiotic.[1] It was also shown that the consumption rate of antibiotics has been 31-35% in hospitalised patients[2] and in our study, 8.6% of patients received antibiotics without any indication or justification. However, the total adherence (appropriateness of antimicrobial agent, initiation time of prophylaxis, duration of prophylaxis, route of prophylactic antibiotic administration) based on the ASHP guideline in 1,000 patients was 0.3%. Moss and colleagues[13] suggested the total rate for inappropriate use of antibiotics up to 47% and the reasons for this inappropriate use were economical factors or clinical errors. Al-Gamadi et al.[11] reported even higher rates of inappropriate usage of antibiotics for prophylactic reasons in the United Arab Emirates as 80% of patients received antibiotics and in 72% of them, the usage was inappropriate.

Overuse of antibiotics together with improper management may cause an increase in direct cost of treatment. Furthermore, overuse and inappropriate use of antibiotics lead to an increase in both bacterial resistance and cost of treatment. Our findings showed that the rate of consuming an antibiotic costs 4.75 times as much as that recommended by ASHP guideline. Because of inappropriate usage, the six hospitals under study paid an extra expense of 54,054,210 Iranian Rials (corresponding to 6,843.26 USD or €5,691.91) for surgical procedures among 1,000 patients.

A number of studies have focused on the issue of increasing cost of antibiotics based on inappropriate usage. Parret et al.[14] showed that by consulting specialists, the cost of antibiotics could decrease by 3.5%. Wilkins and colleagues[15] demonstrated that by consulting specialists only 7% of patients received antibiotics for wrong indications. Furthermore, by consulting infectious disease specialists prior to prescription of antibiotics, Moleski et al.[16] calculated financial savings between 16,000 to 60,000 USD. Hence, consultation and approval of antibiotic prescription by infectious diseases consultants might be an effective option in significant reduction of costs. Also, specific antibiotic training programs showed to be effective in decreasing the frequency of inappropriate usage and costs.[17]

Fraser et al. showed that application of specific guidelines for prescribing antibiotics can lead to an saving up to 400 USD per antibiotic prescription.[18] Pestotnik and colleagues[19] assessed the use of computer based medical expert software for appropriate antibiotic prescription and calculated a 23-25% reduction of costs.

Our study has several limitations. As it is important to mention that there are different factors that affect on an appropriate prescription, including cultural and educational factors, nurses and pharmacists influences, distribution and availability of medical compounds, and logical calculations. The influence of these factors was not examined in our study. In order to ensure cost-effective application of prophylactic antibiotics, Ristic´ et al. showed a significant change in prescribing patterns of antibiotic prophylaxis in caesarean sections following introduction of local guidelines. There was a significant decrease in use of ‘third’ generation of cephalosporin’s whereas the use of ‘‘older’’ antimicrobial drugs with better safety and efficacy increased.[20]

Therefore, we think that it would be necessary to design an evidence based guideline according to local conditions and cultural background in order to improve patient safety and decrease direct costs in the administration of antibiotic prophylaxis to prevent surgical site infection in Shiraz, Iran.
